# The Mitochondrial Permeability Transition Pore in Platelets: Mechanisms, Physiological Roles, and Therapeutic Perspectives

**DOI:** 10.3390/antiox14080923

**Published:** 2025-07-29

**Authors:** Chiara Lonobile, Alessia Di Nubila, Rosa Simone, Matilda Hushi, Silvia Stella Barbieri

**Affiliations:** 1 Unit of Neuro-Cardiovascular Axis, Centro Cardiologico Monzino IRCCS, 20138 Milan, Italy; 2 Department of Biology and Biotechnology “L. Spallanzani”, Università degli Studi di Pavia, Via Bassi 21, 27100 Pavia, Italy; 3 Department of Theoretical and Applied Sciences, eCampus University, 22060 Novedrate, Italy

**Keywords:** platelets, mitochondrial permeability transition pore, therapeutic targets

## Abstract

Platelets have long been known to be critically involved in hemostasis and thrombosis. However, platelets are also recognized as metabolically active cells that require well-regulated mitochondrial function to support their multiple functions in hemostasis, thrombosis, and inflammation. Mitochondrial activity has also recently been shown to play a crucial role in determining platelet activation, survival, and pro-inflammatory potential. A key nexus in these processes is the mitochondrial permeability transition pore (mPTP), a high-conductance channel in the inner mitochondrial membrane. Sustained mPTP opening triggers mitochondrial depolarization, the cessation of ATP synthesis, osmotic swelling, and, finally, platelet dysfunction or clearance. However, its transient opening might play physiological signaling roles. This review summarizes the current understanding of the molecular components and regulatory factors governing the platelet mPTP, explores its physiological and pathological relevance, and evaluates its potential as a therapeutic target in cardiovascular disease, inflammation, cancer, and potentially neurodegenerative diseases. We also highlight the ongoing challenges and crucial future directions in deciphering the complexities of platelet mitochondrial dynamics and mPTP functions.

## 1. Introduction

Platelets are small, anucleate cells derived from megakaryocytes that are important not only for maintaining vascular integrity and primary hemostasis but also for modulating immune responses, inflammation, and tissue remodeling [1,2]. Platelets are metabolically active cells that rely heavily on their intracellular organelles, including mitochondria, to sustain their energy needs and coordinate the signaling pathways that are critical to their activation, aggregation, and secretion [3].

Mitochondria in platelets are not only adenosine triphosphate (ATP) suppliers, they also regulate lifespan and activation [4]. The mitochondrial permeability transition pore (mPTP) is crucial for these mitochondrial functions [5]. The mPTP, traditionally studied in nucleated cells for its role in apoptosis and necrosis, may also have critical effects in the context of the biology of anucleate cells such as platelets [6]. Understanding the platelet mPTP is becoming increasingly important as mitochondrial dysfunction has been linked to platelet hyperactivity in various diseases.

The molecular identity of the mPTP, the channel responsible for this permeability transition, is an area of intense research and debate. While the exact pore composition is controversial, it has been clearly shown, in platelets, that CypD-dependent regulation plays an important role in mPTP dynamics [7].

Opening of the mPTP leads to a sudden loss of mitochondrial membrane potential (ΔΨm), the uncoupling of oxidative phosphorylation, and osmotic swelling and can trigger cell dysfunction or necrotic death [8], which can lead to intrinsic apoptosis [9]. Interestingly, the maintenance of sustained mPTP opening is critical for preventing the “apoptosis-like” phenotype in platelets, suggesting its critical role in the control of programmed cell death in anucleate cells [10]. Furthermore, the sensitivity of platelet mitochondria to calcium overload, oxidative stress, and other stimuli makes the mPTP a pivotal node that translates pathological signals into functional outcomes such as hyperreactivity, exhaustion, or elimination from the circulation [6].

Indeed, the dysregulation of platelet mPTP activity has been identified in a number of clinical conditions, including atherosclerosis, diabetes mellitus, sepsis, malignancies, and neurodegenerative diseases, in which platelet mitochondrial dysfunction and associated oxidative stress contribute to disease pathogenesis [7,11,12,13]. Platelet mPTP is therefore not only a fundamental biological phenomenon but also a promising therapeutic target to selectively modulate pathological platelet activation without profoundly affecting physiological hemostasis.

In this review, we aim to provide a comprehensive and critical overview of the mitochondrial permeability transition pore in platelets. We describe its molecular composition, its activation mechanisms, its physiological and pathological relevance, and the potential to target the mPTP in platelet-centered therapies.

## 2. Biological Background: Platelet Mitochondria

Although platelets are small cell fragments without a nucleus, they have several specialized organelles, including dense granules, alpha-granules, lysosomes, and mitochondria [3]. Typically, each platelet contains 5–8 mitochondria [14], which are strategically located to provide the energy locally required for activation, secretion, shape changes, and thrombus formation [15].

**Figure 1:**
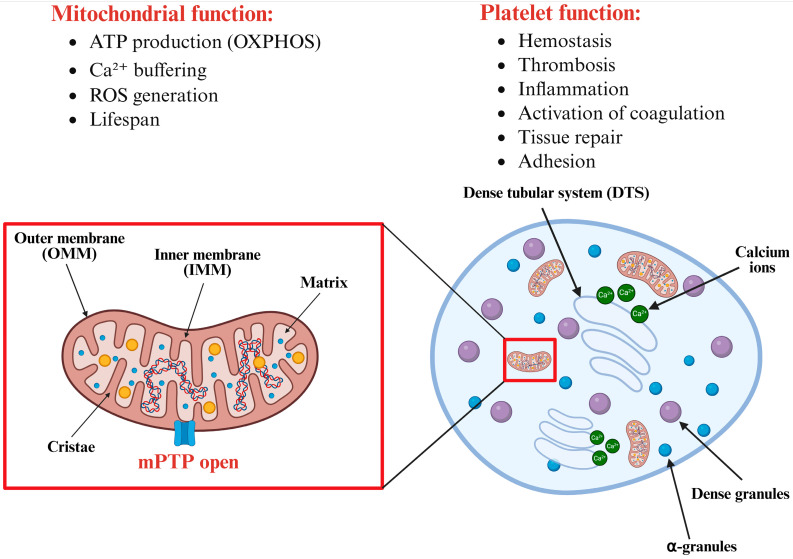
Overview of mitochondrial and platelet functions. Left panel: schematic representation of the main mitochondrial structures—outer membrane (OMM), inner membrane (IMM), cristae, and matrix—and their key functions: ATP production via the TCA cycle and oxidative phosphorylation (OXPHOS), buffering of intracellular calcium, generation of reactive oxygen species (ROS), and regulation of platelet lifespan. The opening of the mitochondrial permeability transition pore (mPTP) is a critical event that can lead to the loss of mitochondrial membrane potential (ΔΨm), ROS accumulation, and the initiation of apoptotic-like signaling pathways. Right panel: schematic overview of key platelet organelles and functions, including mitochondria, alpha-granules, dense granules, and the dense tubular system (DTS). The DTS, analogous to the endoplasmic reticulum, plays a key role in calcium storage and signaling and closely interacts with mitochondria to regulate calcium dynamics. Together, these organelles support essential platelet functions such as hemostasis, thrombosis, inflammation, activation, tissue repair, and adhesion. Created with BioRender.com.

Platelet mitochondria have more than just a bioenergetic function and produce ATP through the tricarboxylic acid (TCA) cycle and oxidative phosphorylation (OXPHOS) [16]. Although resting platelets mainly rely on glycolysis for energy requirements, mitochondrial ATP production is crucial during activation or under hypoxia [17]. In addition, mitochondria play fundamental non-energetic roles that are crucial for platelet function and survival as they are involved in intracellular calcium homeostasis [18], reactive oxygen species (ROS) generation [19], and apoptotic-like signaling [10]. These mitochondrial functions, however, are not unique to platelets, but rather reflect the conserved mechanisms shared across many cell types. Mitochondria also communicate extensively with other organelles, particularly the dense tubular system (an analogue of endoplasmic reticulum -ER-), at contact sites that are critical for effective calcium transfer and therefore can potentially modulate mPTP sensitivity [20]. An overview of mitochondrial and platelet functions is summarized in Figure 1.

The electrical potential at the inner mitochondrial membrane (ΔΨm) serves an important indicator of the healthy state of mitochondria. In fact, the maintenance of ΔΨm is crucial for the function of the electron transport chain (ETC) and for the production of ATP. Changes in ΔΨm, often due to the opening of mPTP, are an early indicator of mitochondrial dysfunction, which can lead to an apoptotic-like platelet phenotype characterized by the exposure of phosphatidylserine (PS) on the outer membrane, the shedding of microparticles, and their eventual clearance from the circulation [6,10].

Interestingly, although platelets lack nuclear transcription, they have mitochondrial DNA (mtDNA) and mitoribosomes. Alterations in mtDNA affect the mitochondrial translational capacity, potentially leading to platelet dysfunction [21]. This ability may allow platelets to respond dynamically to stress by modulating the mitochondrial protein concentration, although the extent and significance of this process are the subject of active investigation.

Mitochondria also interact with cytosolic signaling pathways. During activation, mitochondrial ROS (mtROS) act as secondary messengers that amplify signals downstream of receptors such as glycoprotein VI (GPVI) and integrins [7,14]. In addition, calcium influx triggered by agonists such as thrombin or collagen leads to mitochondrial calcium uptake via the mitochondrial calcium uniporter (MCU), which in turn regulates both mitochondrial metabolism and sensitivity to permeability transitions [22].

Age-related changes in platelet mitochondria are also well-documented. Older platelets show a decrease in mitochondrial function, increased ROS production, reduced ΔΨm, and enhanced sensitivity to mPTP opening [23]. It is speculated that this mitochondria decline contributes to platelet clearance and lifespan. Since the mitochondria turnover rate in many nucleated cells (9–12 days) [24] is longer than the platelet lifespan (7–10 days) and platelets cannot efficiently replenish mitochondrial proteins, it can be assumed that the functional longevity of mitochondria influences platelet lifespan.

In summary, platelet mitochondria serve as central hubs for bioenergetic support, intracellular signaling, and the modulation of life expectancy. Given their integral role, mitochondrial dysfunction, particularly through the dysregulation of mPTP opening, can affect platelet physiology and contribute to pathological conditions.

## 3. Mitochondrial Permeability Transition Pore (mPTP) in Platelets

The mPTP is a non-specific high-conductance channel that can form in the inner mitochondrial membrane under pathological conditions. Its opening leads to a balance of ions and small solutes (<1.5 kDa) in the inner membrane, resulting in a rapid loss of ΔΨm, swelling of the matrix, the uncoupling of OXPHOS, and eventually rupture of the outer mitochondrial membrane [5]. Persistent mPTP opening is an important factor for necrosis and apoptosis in nucleated cells. In platelets, the mPTP plays similarly crucial but distinct roles due to their anucleate nature and unique mitochondrial physiology [7,25].

### 3.1. Molecular Composition and Regulation

**Figure 2:**
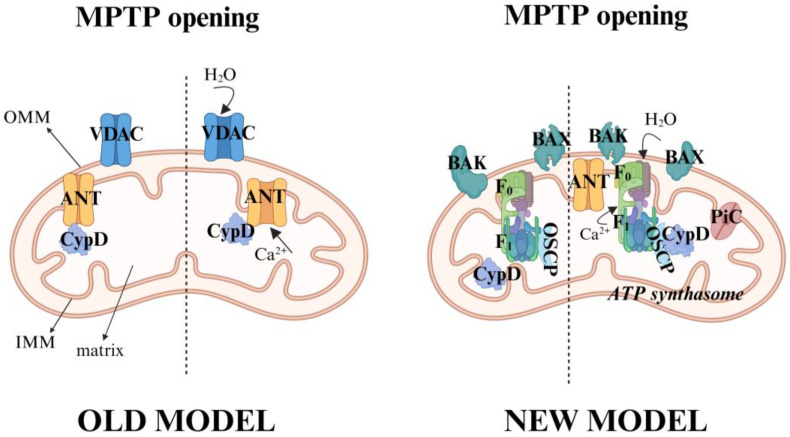
Molecular models and regulation of the mitochondrial permeability transition pore (mPTP). The figure shows the proposed mechanisms for the opening of the mPTP. In the old model, the pore is formed by the interaction of the voltage-dependent anion channel (VDAC), located on the outer mitochondrial membrane (OMM); the adenine nucleotide translocase (ANT), located on the inner mitochondrial membrane (IMM), and the matrix protein Cyclophilin D (CypD), located in the matrix, which interact to form the pore. On the contrary, the new model, known as the ‘ATP synthasome’, questions the involvement of VDAC and ANT, as essential pore components and suggests that other mitochondrial proteins, such as F_1_ F_0_ subunits of the ATP synthase, located in the IMM, are key structural components to the pore. CypD plays a regulatory role by interacting with other subunits, such as the Oligomycin-Sensitivity-Conferring Protein (OSCP). In particular, the current consensus is that CypD is a calcium and ROS regulator. Created with BioRender.com.

The exact molecular identity of the mPTP is one of the most debated topics in mitochondrial biology and is not yet fully understood. In the past, a multiprotein complex of mitochondrial membranes including inner membrane proteins such as adenine nucleotide translocase (ANT) [26,27], outer membrane proteins such as the voltage-dependent anion channel (VDAC) [28,29], and the peripheral benzodiazepine receptor (TSPO), along with the matrix protein Cyclophilin D (CypD) [30,31] has been proposed. However, genetic studies (primarily in non-platelet cells) have shown that mitochondria lacking ANT and/or VDAC can still undergo a permeability transition, challenging their role as essential pore components [26,28]. The current consensus is that CypD is a critical calcium- and ROS-sensitive regulator [7,32,33]. It is hypothesized that upon binding to its targets (potentially involving ANT, the phosphate carrier, or other inner membrane proteins, which is still under debate), CypD triggers a conformational change that promotes pore opening [32,34,35]. Figure 2 shows both the old and new models proposed for mPTP pore formation.

Crucially, functional studies on platelets confirm this essential regulatory role of CypD [7]. Platelets isolated from CypD-deficient mice exhibit increased resistance to the calcium-induced permeability transition, low ROS production, and prolonged survival under stress conditions [7]. Accordingly, pharmacological inhibitors of CypD, such as cyclosporin A (CsA) and its non-immunosuppressive analogues, prevent the opening of mPTP and protect platelet mitochondrial function during oxidative or metabolic insults [7,10,22,36]. The potential regulatory involvement of TSPO remains less clear and possibly context-dependent [32,33].

Recent evidence suggests that other mitochondrial proteins, including subunits of the F_1F_0-ATP synthase complex, particularly the c-subunit ring, may contribute to mPTP formation under specific conditions [5,37]. Whether this model fully applies to platelets requires further specific investigations.

In addition to these proposed core components and regulators, mPTP sensitivity is modulated by several factors including the matrix pH, Mg^2+^ levels, adenine nucleotide concentration, inorganic phosphate, and the redox state of specific protein thiols [25,32]. Although the exact structure is elusive and likely dynamic, CypD is the best-established regulatory target for mPTP modulation in platelets.

### 3.2. Triggers of mPTP Opening in Platelets

**Figure 3:**
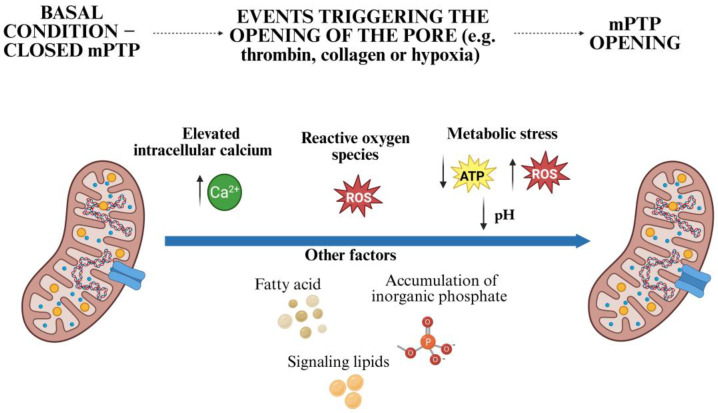
Mechanisms leading to sustained opening of the mitochondrial permeability transition pore (mPTP) in platelets. The figure is organized in three panels to illustrate the dynamic progression of mPTP opening. Under resting conditions (left panel) the mPTP remains closed. Upon stimulation by platelet activators (e.g., thrombin, collagen) or energetic stress (e.g., hypoxia) (middle panel), increased cytosolic Ca^2+^, elevated ROS levels, and ATP depletion lead to calcium entry. These stimuli contribute to the opening of the mPTP (right panel). Created with BioRender.com.

Several stimuli can trigger mPTP opening in platelets, often through a combination of mitochondrial calcium overload and oxidative stress (Figure 3). The most important triggers include the following:

(a) Elevated intracellular calcium: platelet activation by agonists such as thrombin, collagen, or adenosine diphosphate (ADP) leads to a rapid increase in cytosolic calcium, some of which is sequestered in the mitochondria via the MCU. Excessive mitochondrial calcium accumulation promotes opening of the mPTP [22,38].
(b) Reactive oxygen species (ROS): the formation of ROS, generated by mitochondrial ETC and NOX enzymes, can oxidize mitochondrial proteins and lipids (e.g., cardiolipin) and lower the calcium threshold for mPTP opening [7,22].
(c) Persistent metabolic stress: pathological conditions such as ischemia reperfusion or sepsis lead to ATP depletion, intracellular acidosis, and further ROS production, which promotes the opening of the mPTP [32].
(d) Other Factors: fatty acids, the accumulation of inorganic phosphate, and potentially certain signaling lipids can also sensitize the pore [25].

### 3.3. Unique Aspects of mPTP Behavior in Platelets

In contrast to nucleated cells, in which sustained mPTP opening often leads to necrosis or classical apoptosis with nuclear fragmentation, mPTP opening in platelets causes a form of “anucleate programmed cell death” characterized by the following:

(a) mitochondrial depolarization: the rapid collapse of ΔΨm is a hallmark of mPTP opening [7].
(b) phosphatidylserine (PS) exposure: the externalization of PS on the platelet surface serves as an “eat-me” signal for clearance by macrophages [10,22,39].
(c) procoagulant microparticles generation: platelets undergoing mPTP-mediated apoptosis can release microparticles rich in PS and tissue factor that enhance the thrombotic potential [40].

Furthermore, there is intriguing evidence that transient, reversible mPTP opening (“flickering”) can be observed during physiological platelet activation, which may serve as a signaling event rather than a death trigger [6]. This emphasizes the complex and context-dependent role of mPTP in platelets. Distinguishing between the mechanisms that control transient and sustained opening remains a key challenge. However, emerging evidence suggests that the intensity and duration of mitochondrial calcium influx, along with local ROS levels and the cellular ATP/ADP ratio, may play a central role in this regulation. Additionally, the activity of CypD, a known mPTP regulator, may be modulated through post-translational modifications such as phosphorylation or acetylation, which could influence the pore’s sensitivity to stress signals. Moreover, the interactions between mitochondria and the dense tubular system DTS, through which calcium microdomains are tightly regulated, can tune the threshold for pore flickering versus full opening.

Such control mechanisms may allow platelets to exploit short-lived mPTP openings as a signaling mechanism (e.g., ROS bursts for activation), while preventing irreversible mitochondrial dysfunction. These dynamic properties highlight the dual role of the mPTP as both a metabolic integrator and a molecular switch in platelet fate.

## 4. Mechanisms of mPTP Opening in Platelets

The regulation of mPTP opening in platelets is a highly coordinated process that integrates signals from the metabolic status, calcium dynamics, oxidative stress, and the post-translational modifications of mitochondrial proteins. Understanding these mechanisms is crucial, as inappropriate mPTP activation can lead to pathological platelet responses.

### 4.1. Mitochondrial Calcium Overload

Calcium ions are pivotal regulators of mPTP opening. Platelets have a finely tuned system for intracellular calcium mobilization during activation, which includes release from intracellular stores (dense tubular system) and influx from the extracellular milieu through store-operated calcium entry (SOCE) mechanisms [41,42]. Mitochondria act as transient buffers that rapidly take up cytosolic calcium via the MCU complex [43,44].

Under physiological conditions, mitochondrial calcium accumulation supports ATP production by activating the dehydrogenases of the TCA cycle. However, the accumulation of large amounts of calcium in the mitochondrial matrix leads to the opening of the mPTP, especially under oxidative stress conditions or energy deficiency [32]. The pharmacological inhibition of MCU prevents mitochondrial calcium overload and reduces mPTP opening, resulting in the preservation of platelet function under stress conditions in experimental models [45].

### 4.2. Oxidative Stress and Reactive Oxygen Species (ROS)

ROS generation is another critical modulator of mPTP dynamics. Platelet activation leads to the production of ROS mainly by complexes I and III of the mitochondrial ETC and potentially from cytosolic sources as NOX complexes [46]. ROS can oxidize critical thiol residues on proteins such as CypD and ANT, lowering the threshold for pore opening [5,32,47]. In addition, ROS can oxidize mitochondrial membrane lipids, particularly cardiolipin, destabilizing the inner membrane structure and the integrity of the respiratory chain complexes, thereby promoting the permeability transition [48,49]. Consequently, the inhibition of mitochondrial ROS by antioxidants (e.g., the mitochondria-targeted antioxidant MitoTEMPO) or the overexpression of mitochondrial antioxidant enzymes (e.g., manganese superoxide dismutase, MnSOD) reduce mPTP-dependent platelet dysfunction in different experimental settings [7,10,39,50].

### 4.3. Role of Cyclophilin D (CypD)

CypD, a mitochondrial peptidyl-prolyl isomerase, acts as an important gatekeeper for mPTP opening. In response to stress signals such as increased matrix calcium and/or oxidative stress, CypD binds to inner membrane components (or potentially the F-ATP synthase), enabling a conformational change that promotes pore opening [32,34,35]. Its genetic ablation or pharmacological inhibition (e.g., with CsA or its non-immunosuppressive derivatives such as sanglifehrin A) leads to resistance to mPTP opening and protects platelet mitochondrial function [4,7].

Interestingly, the activity of CypD is modulated by post-translational modifications such as acetylation. The deacetylation of CypD by sirtuin 3 (SIRT3), a mitochondrial NAD^+^-dependent deacetylase, suppresses mPTP opening, suggesting that the metabolic status (reflected in NAD^+^ levels) may influence platelet survival by regulating CypD [51].

### 4.4. Energetic Stress and ATP Depletion

Mitochondrial function is intrinsically linked to the energy status of the cells. Energetic failure, characterized by reduced mitochondrial ATP production and a drop in the ATP/ADP ratio, sensitizes the mPTP to open [25,32]. ATP depletion impairs mitochondrial ion homeostasis (e.g., by affecting proton pumping and K^+^ channels) and promotes membrane depolarization, creating conditions for the mPTP-mediated permeability transition. This mechanism is particularly important in pathological conditions such as myocardial infarction, where platelets are exposed to a hypoxic and ischemic environment.

### 4.5. Involvement of the F_1_F_0_-ATP Synthase Complex

As mentioned above, recent studies suggest that the c-subunit ring of the F_1_F_0_-ATP synthase complex may function as a structural component of mPTP under certain pathological conditions [32,37]. A dysfunctional ATP synthase, especially when hyperphosphorylated or oxidized, could undergo conformational changes that convert the enzyme into a pore-forming structure. Although this hypothesis has primarily been investigated in nucleated cells, recent data suggest that similar mechanisms may also operate in platelets, warranting further investigation.

## 5. Physiological and Pathological Roles of mPTP in Platelets

**Figure 4:**
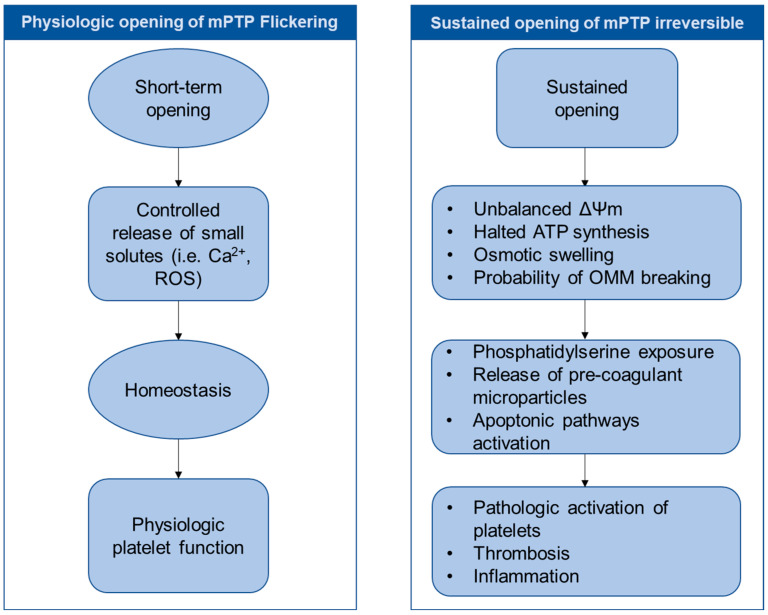
Flow diagram of the physiological and pathological consequences of mPTP Opening. Under physiological conditions, the transient and regulated opening of the mPTP may allow the uncontrolled release of small molecules or ions, such as calcium or reactive oxygen species (ROS), from the matrix into the cytosol. This process could contribute to maintaining homeostasis and thereby ensuring proper platelet physiological function. In contrast, sustained and irreversible mPTP opening leads to mitochondrial disfunction, characterized by a collapse of the mitochondrial membrane potential (ΔΨm), the halt of ATP synthesis, osmotic swelling, and the probability of the outer mitochondrial membrane (OMM). Sustained and irreversible mPTP opening also results in phosphatidylserine exposure, the release of pro-coagulant microparticles, and the activation of apoptotic pathways. The dysregulation of mPTP opening can lead to pathological platelet activation, thereby promoting thrombosis and inflammation.

The mPTP plays a crucial role in platelet function and influences several processes, from activation and survival to clearance and hyperreactivity under pathological conditions. Its activity must be carefully regulated to maintain the balance between hemostasis and thrombosis (Figure 4).

### 5.1. Physiological Roles: Beyond Cell Death Signaling?

Under physiological conditions, the transient and regulated opening of the mPTP (“flickering”) could serve as a signaling mechanism rather than a trigger for irreversible damage. These transient openings could allow the uncontrolled release of small molecules or ions (e.g., calcium and ROS) from the matrix into the cytosol, potentially controlling signaling pathways involved in platelet activation, managing mitochondrial calcium loading or redox balance adjustments [32,37]. This transient activity could help fine-tune platelet responses during activation, ensure adequate energy production, and maintain a healthy redox balance.

In addition, the mild, transient depolarization of mitochondria through this transient mPTP opening could prevent excessive calcium accumulation and ROS production, thus protecting platelets from premature dysfunction [2]. It has been suggested that in normal responses, a dynamic mitochondrial response, including regulated mPTP activity, supports sustained platelet function without triggering pro-apoptotic pathways [6]. It may also provide a mechanism to reduce excessive mitochondrial polarization under certain conditions. However, the exact nature and function of physiological mPTP flickering specifically in platelets is still poorly understood and technically difficult to study directly.

### 5.2. mPTP Opening and Platelet Clearance

Sustained or irreversible mPTP opening leads to profound mitochondrial depolarization, ATP depletion, and the initiation of an apoptosis-like process:

(a) Mitochondrial disfunction: structural changes in mitochondria, the collapse of ΔΨm, ATP depletion, and increased ROS production signal irreversible damage [7]. Swelling and fragmentation of the mitochondria are also evident.
(b) phosphatidylserine (PS) exposure: the subsequent externalization of PS on the surface of platelets marks them for recognition and clearance by macrophages via scavenger receptors, which remove senescent or damaged platelets from the circulation [10,39].
(c) Microparticle shedding: platelets undergoing apoptosis-like changes can release microparticles that are enriched in procoagulant lipids and proteins and can contribute to thrombin generation [40].

This process is crucial for the clearance of aged or damaged platelets, thereby maintaining platelet homeostasis and preventing the accumulation of dysfunctional, potentially prothrombotic platelets.

### 5.3. Pathological Effects of Dysregulated mPTP Opening

The dysregulation of mPTP opening, which often occurs with excessive oxidative stress and/or calcium overload, has been identified in several pathological conditions in which platelet mitochondrial dysfunction contributes to disease progression:

(a) Cardiovascular Diseases: In acute coronary syndromes (ACSs) and atherosclerosis, platelets exhibit increased mitochondrial ROS production, enhanced sensitivity to mPTP opening, and a procoagulant phenotype [6]. This increased mPTP activity promotes platelet hyperreactivity, contributes to thrombosis and can favor vascular occlusion. Targeting mPTP opening with pharmacological agents has shown promise in experimental models to reduce thrombus formation without impairing basal hemostasis [52].
(b) Diabetes Mellitus: Platelets from patients with diabetes have mitochondrial dysfunction characterized by increased ROS, mitochondrial calcium overload, and a lower threshold for mPTP opening [53]. These mitochondrial abnormalities contribute to platelet hyperactivity, exacerbate endothelial damage, and may accelerate the development of vascular complications.
(c) Sepsis: In sepsis, systemic inflammation and oxidative stress have a strong impact on platelet mitochondria. Enhanced mPTP opening can lead to platelet exhaustion, increased clearance, and thrombocytopenia and contributes to disseminated intravascular coagulation (DIC) through the release of procoagulant microparticles [54]. Paradoxically, the initial hyperactivation of platelets in sepsis may also be related to mitochondrial signaling before the onset of exhaustion.
(d) Neurodegenerative Diseases: Emerging evidence links systemic mitochondrial dysfunction to neurodegenerative disorders such as Alzheimer’s and Parkinson’s disease. Platelet mitochondrial dysfunction, including dysregulated mPTP activity and altered redox state, may serve as a biomarker for systemic oxidative stress and mitochondrial impairment in these diseases [55].
(e) Cancer and Inflammation: Platelets play a complex role in carcinogenesis (e.g., promoting metastasis) and in chronic inflammatory diseases. Research is ongoing but mitochondrial function, redox signaling, and potentially mPTP dysregulation likely influence platelet interactions with tumor and immune cells, contributing to these pathologies [56]. Targeting platelet mitochondria could offer new therapeutic opportunities in these contexts.

## 6. Targeting the mPTP in Platelets: Therapeutic Opportunities

Given the central role of the mPTP in linking mitochondrial stress (calcium overload, oxidative damage, energy deficiency) to platelet activation, survival, and clearance, therapeutic strategies targeting the modulation of mPTP have emerged as attractive therapeutic targets. The pharmacological inhibition of pathological mPTP opening has the potential to reduce the risk of thrombosis, alleviate inflammation, and preserve physiological platelet function without severely impairing physiological hemostasis. Agents targeting CypD or increasing mitochondrial resistance are currently being investigated to prevent pathological mPTP opening without impairing platelet physiological functions [52,57,58,59,60,61].

### 6.1. Cyclophilin D Inhibitors: The Leading Strategy

Due to its role in mPTP opening, CypD is a primary therapeutic target.

(a) Cyclosporin A (CsA): CsA inhibits mPTP opening by binding to CypD and preventing its interaction with putative pore components, thereby stabilizing the mitochondrial inner membrane and preserving mitochondrial integrity [62]. This preserves ΔΨm, limits ATP depletion and reduces the release of pro-apoptotic factors. CsA can also enhance the mitochondrial calcium-buffering capacity in several diseases [63].
  Preclinical studies support the cardioprotective effect of CsA and its analogue Sanglifehrin A in an ischemic myocardial infarction model [59,64,65,66,67,68]. In addition, CsA reduces the liver damage after ischemia–reperfusion injury [69], mitigates the stroke outcome, and improves mitochondrial function after transient middle cerebral artery occlusion [70]. However, a meta-analysis of randomized controlled trials showed that the administration of CsA does not protect the heart from reperfusion injury in clinical patients with myocardial infarction [71]. This non-effect may be related to its controversial activity on platelets.
  It has been reported that CsA can enhance platelet procoagulant activity, intracellular calcium mobilization, and platelet aggregation in response to ADP [72,73]. In contrast, others showed a protective effect of CsA on platelet apoptosis, reduced PS exposure, and the loss of ΔΨm induced by strong agonists such as collagen and thrombin [10,39,74].
  Recently, a potential link between cellular metabolism and mPTP regulation has been uncovered [43]. Platelets rely heavily on both aerobic glycolysis and mitochondrial oxidative phosphorylation for energy production, and perturbations in these metabolic pathways can affect mitochondrial dynamics and functions. In particular, specific mitochondrial and glycolytic enzymes may modulate mPTP opening either directly, through structural interactions, or indirectly by altering the redox balance, calcium handling, or nucleotide availability (ATP/ADP ratio).
  This suggests that metabolic reprogramming could be a novel avenue for the therapeutic modulation of mPTP activity. Agents targeting key metabolic enzymes or regulators such as hexokinase II, pyruvate dehydrogenase, or mitochondrial complex I may provide a dual benefit by preserving mitochondrial function and reducing platelet hyperactivation. This area remains poorly understood in the context of platelet physiology. However, its potential to influence the interactions between CypD and mPTP through metabolic control should be further investigated as a complementary strategy to direct mPTP inhibition.
(b) Non-immunosuppressive CsA derivatives: Agents such as Debio-025 and NIM811, analogs of CsA, have a CypD-inhibiting effect without significant immunosuppression. They have shown promise in preclinical models by reducing the ischemia–reperfusion injury damage during hepatic surgery [69], as well as brain and heart damage post-cardiac arrest [75,76,77]. Their direct effect on platelet function is poorly studied [78]; some studies mention a reduction in platelet counts and the modulation of platelet-derived growth factor receptors after treatment with Debio-025 [79]. These compounds are currently being investigated for their ability to protect platelet mitochondria in cardiovascular and metabolic diseases.
(c) Molecules unrelated to CsA: Recently, novel small-molecule CypD inhibitors (F759 and F83236) that are structurally unrelated to CsA have been developed. These agents promote the loss of mitochondrial membrane potential, reduce procoagulant platelet formation and the clotting time, and reduce fibrin formation when stimulated by dual-agonists (convulxin plus thrombin) without modifying P-selectin expression and integrin αIIbβ3 activation. In contrast to CsA, they did not enhance ADP-induced platelet aggregation [80].
(d) Sirtuin 3 (SIRT3) activators: Enhancing endogenous protective mechanisms represents an additional strategy. SIRT3, a mitochondrial NAD^+^-dependent deacetylase, inhibits mPTP opening by deacetylating CypD at lysine 166 [51]. Compounds such as honokiol, a natural polyphenol from the Magnolia plant, activate SIRT3 and have shown protective effects in preclinical cardiac and neuronal models by improving mitochondrial function and reducing oxidative stress [81]. Although direct studies on platelet activation by honokiol are lacking, affecting SIRT3 activity could theoretically stabilize mitochondrial function in platelets, reduce oxidative stress, and prevent inappropriate platelet activation. Further research is needed to clarify the specific effects of SIRT3 agents such as honokiol on platelet function.

### 6.2. Compounds Binding to the Translocator Protein (TSPO) Located on the Outer Mitochondrial Membrane

Synthetic ligands such as TRO40303 and TRO19622, which exert their effect by binding to the TSPO on the outer mitochondrial membrane, have been synthetized [82,83,84]. TRO40303 showed promise in preclinical models of ischemia–reperfusion injury, reducing infarct size [84], with safety and tolerability in a randomized Phase I trial [85]. However, it failed to show efficacy in a Phase II clinical trial on patients with myocardial infarction [86].

### 6.3. Mitochondria-Targeted Antioxidants: Combating Oxidative Stress

As oxidative stress is an important sensitizer for mPTP opening, directly targeting mitochondrial ROS (mROS) is a relevant strategy.

(a) Mitochondria-targeted antioxidants: Compounds such as MitoTEMPO, an SOD mimetic, or SkQ1 and MitoQ, which accumulate in the mitochondrial matrix, reduce mitochondrial ROS levels and have been shown to protect platelet function under oxidative stress both in vitro and in vivo [87,88,89].
(b) Natural antioxidants: Several natural compounds with antioxidant properties inhibit platelet activation, potentially via mechanisms involving mPTP modulation. **Icariin** and **gallic acid**, which are known to prevent the downstream activation of mPTP [60,61,90], decrease ROS production and platelet activation [91,92]. In addition, a **selenium-containing protein** from selenium-enriched *Spirulina platensis* counteracts oxidative damage by regulating mPTP opening [93]. Finally, **Schisandrin B**, a lignan from *Schisandra chinensis*, inhibits mPTP opening and preserves cardiomyocytes from anthracycline-induced cardiotoxicity by maintaining mitochondrial integrity and reducing oxidative stress [94,95].

### 6.4. Modulators of Mitochondrial Calcium Handling: Reducing the Trigger

Since excessive mitochondrial calcium uptake is an important trigger for mPTP opening, strategies targeting calcium handling are relevant:

(a) MCU inhibitors: The direct pharmacological blockade of MCU (e.g., Ru360 or newer small molecules) can attenuate calcium-induced mPTP opening in isolated mitochondria and cells [96,97]. Despite these promising experimental approaches, the systemic effects of MCU inhibition need to be carefully evaluated due to the fundamental role of mitochondrial calcium in cellular metabolism. A major translational challenge is the limited membrane permeability of classical MCU inhibitors such as Ru360 [98], which significantly hampers their clinical applicability.
  Newer compounds such as Ru265 show improved cell permeability and efficacy in preclinical models, particular a reduction in ischemic brain injury at low doses. However, high doses have been associated with severe side effects such as fatal convulsions [99]. Although promising, the safety and therapeutic window of this drug in vivo remain to be well defined. These limitations need to be considered when evaluating the feasibility of MCU as a therapeutic target.
(b) Indirect modulation via cytosolic calcium: An alternative strategy to prevent mPTP opening is the indirect modulation of mitochondrial calcium uptake by targeting cytosolic calcium entry mechanisms. In particular, store-operated calcium entry (SOCE), which is mediated by STIM1-Orai1, is crucial for maintaining platelet calcium influx after depletion of the intracellular calcium store. Excessive SOCE activity during pathological activation contributes to mitochondrial calcium overload, which promotes mPTP opening and downstream platelet dysfunction. The genetic or pharmacological inhibition of STIM1 or Orai1 impairs platelet calcium signaling, reduces aggregation, and protects against thrombotic events, supporting the therapeutic potential of targeting these channels to prevent calcium-induced mitochondrial dysfunction in platelets [100,101,102,103]. The pharmacological inhibition of SOCE channels such as BTP-2 or SKF-96365 attenuates the mitochondrial calcium increase, preserves ΔΨm, and reduces platelet activation [104,105].

### 6.5. Modulators of the F_1_F_0_-ATPase: Enhancing Mitochondrial Function

Emerging evidence suggests that the F_1_F_0_-ATPase complex is involved in mPTP, making it a potential target.

(a) Inhibitors potentially acting via ATP synthase modulation: 1,2,3-triazole derivatives inhibit platelet aggregation induced by ADP or collagen [106], and 1,5-disubstituted-1,2,3-triazoles attenuate mPTP opening and reduce oxidative stress, thereby protecting cardiovascular cells from damage [107,108].
  new class of inhibitors of the F_1_F_0_-ATPase complex, the 1,3,8-Triazaspiro [4.5] decane derivatives, targeting the c subunit, reduced myocardial reperfusion injury [106], but their effects on platelet function, to our knowledge, are unknown. The quinoline-4-carboxamide ER-000444793, identified as an inhibitor of the mPTP complex [109], was mainly studied in vascular smooth muscle cells [110].
(b) Natural compounds: Naringenin, a flavonoid, modulates mPTP opening, preserves cerebral endothelial cells in spontaneously hypertensive-stroke-prone rats (SHRSP) [111], and inhibits platelet activation and thrombus formation in in vitro and in vivo mouse models [112].
  Finally, melatonin, known for its antioxidant properties and platelet-inhibitory effects [113,114], can rescue impaired mitochondrial respiration by targeting the F_1_F_0_-ATPase complex in aortic endothelial cells [115].

### 6.6. Challenges and Future Directions: Moving Towards the Clinic

While preclinical data suggest that targeting the mPTP is a viable strategy, several challenges remain. Specifically, therapeutic agents must selectively modulate pathological mPTP opening without interfering with physiological mitochondrial signaling and platelet functions. Since mitochondria are ubiquitous, systemic mitochondrial modulation carries the risk of off-target effects in tissues other than platelets. Most agents targeting mPTP compounds are in early-phase trials or preclinical stages; robust clinical data in patients with thrombotic or inflammatory diseases are still lacking.

Despite promising preclinical data, the translation of mPTP modulation into safe and effective therapies faces major hurdles:

Selectivity: Therapeutic agents must preferentially inhibit pathological, sustained mPTP opening without interfering with essential physiological mitochondrial functions (such as transient signaling or ATP production) or the basal platelet activity required for hemostasis. The concept of “pathological vs. physiological” mPTP opening needs to be more clearly defined at the molecular level.

Off-Target Effects: Mitochondria are ubiquitous. The systemic administration of mPTP modulators carries a substantial risk of affecting mitochondria in other vital organs (heart, brain, liver, kidney). The development platelet-specific delivery approaches or the identification of therapeutic windows in which platelet mPTP sensitivity differs from other tissues is crucial.

Clinical relevance: Most mPTP-targeted agents are not yet used in the clinic. Rigorous clinical studies demonstrating the efficacy and safety of MG in platelet-induced dysfunction (thrombosis, inflammation) are largely missing.

Biomarkers: Specific biomarkers to measure platelet mitochondrial health and mPTP status in vivo would be crucial for patient stratification and therapeutic-response monitoring. Platelet mPTP sensitivity could fulfil this role.

The following fundamental questions remain:

- What is the definitive molecular composition of platelet mPTP, and does it vary under different pathological conditions or between individuals?
- What are the precise molecular switches and post-translational modifications that distinguish transient, potentially physiological flickering, from persistent, pathological pore opening?
- How do mitochondrial dynamics (fusion/fission events) and interorganelle communication (e.g., with ER/DTS) overlap with mPTP regulation in platelets?
- What are the most reliable and feasible methods to study mPTP dynamics (e.g., flickering vs. full opening) in circulating human platelets or in relevant in vivo models?

Addressing these challenges requires continuous multidisciplinary research combining molecular biology, pharmacology, and clinical investigations (Table 1).

## 7. Conclusions

The mitochondrial permeability transition pore in platelets has evolved from a specialized research topic to a central regulator of platelet biology and pathobiology. Platelet mitochondria, in which the mPTP acts as a critical checkpoint, are now much more than simple energy producers; they are important regulators of platelet activation thresholds, signaling pathways, the lifespan, and clearance mechanisms. While sustained mPTP opening, often triggered by excessive calcium influx and/or oxidative stress, leads to platelet dysfunction, hyperactivation, or the apoptosis-like death that contributes to thrombosis and inflammation, there is increasing evidence that transient mPTP activity may fulfil physiological signaling or quality control functions.

Although the exact molecular nature of the pore is still under investigation, key regulatory factors such as CypD and important triggers such as calcium and ROS are well understood and offer tangible therapeutic targets. The pharmacological modulation of mPTP with specific CypD inhibitors, mitochondria-targeted antioxidants, or potential agents that modulate the calcium balance or ATP synthase function represents an exciting therapeutic avenue for cardiovascular, inflammatory, and potentially other diseases in which platelet dysfunction plays a key role. However, significant challenges need to be overcome in terms of selectivity, potential off-target effects, and the need for robust clinical validation.

Future research should focus on deciphering the exact molecular intricacies of platelet mPTP, clearly distinguishing its physiological and pathological functions, developing more targeted and safer therapeutic strategies, and establishing reliable biomarkers for clinical application. Unravelling the remaining mysteries of platelet mPTP holds significant potential for the development of novel treatments for a variety of debilitating human diseases.

## Figures and Tables

**Table 1:** Therapeutic strategies targeting platelet mPTP.

Therapeutic Strategy	Primary Target	Mechanism of Action	Examples	Advantages	Limitations/Challenges
**Cyclophilin D (CypD) Inhibitors**	CypD	Prevents mPTP opening by binding CypD	Cyclosporin A (CsA), Sanglifehrin A	Mitochondrial protection; reduced ischemic damage	Immunosuppression, limited long-term use; ineffective in clinical trials; potential pro-thrombotic effects
		Non-immunosuppressive inhibition of CypD	Debio-025, NIM811	Avoids immunosuppression; reduces ischemia–reperfusion damage	Platelet effects poorly characterized; limited clinical validation
		Novel small-molecule CypD inhibitors (CsA-unrelated)	F759, F83236	Reduce platelet procoagulant activity without affecting integrin signaling	Still experimental; lack of human data
	SIRT3	Promotes CypD deacetylation and inhibits mPTP	Honokiol	Neuroprotective and cardioprotective; reduces oxidative stress	No direct data on platelets; potential systemic off-target effects
**TSPO Ligands**	Translocator Protein (TSPO)	Stabilizes mitochondrial membranes	TRO40303, TRO19622	Effective in reducing infarct sizes in animal models	Failed efficacy in Phase II trials; unclear mitochondrial specificity
**Mitochondria-Targeted Antioxidants**	Mitochondrial ROS	Scavenge ROS within mitochondria	MitoTEMPO, SkQ1, MitoQ	Improves mitochondrial function; prevents platelet dysfunction under oxidative stress	Long-term safety in humans unclear; dose optimization needed
		Natural antioxidants modulating mPTP and reducing ROS	Icariin, Gallic Acid, Schisandrin B, Spirulina protein	Natural origin; multiple antioxidant and antiplatelet effects	Variability in bioavailability and formulation; limited clinical data
**Modulation of Mitochondrial Calcium Handling**	MCU (Mitochondrial Calcium Uniporter)	Inhibits mitochondrial calcium uptake to prevent mPTP	Ru360, other MCU blockers	Reduces calcium-triggered mitochondrial dysfunction	May impair essential metabolic processes regulated by calcium
	SOCE (STIM1-Orai1)	Limits cytosolic calcium entry and mitochondrial overload	BTP-2, SKF-96365	Reduces platelet activation and thrombotic risk	Risk of systemic immunosuppression; lacks platelet-specific targeting

## Abbreviations

The following abbreviations are used in this manuscript:

ACS	acute coronary syndromes
ADP	adenosine diphosphate
ANT	adenine nucleotide translocase
ATP	adenosine triphosphate
CsA	cyclosporin A
CypD	Cyclophilin D
DIC	disseminated intravascular coagulation
ER	endoplasmic reticulum
ETC	electron transport chain
GPVI	glycoprotein VI
ΔΨm	mitochondrial membrane potential
MCU	mitochondrial calcium uniporter
MnSOD	manganese superoxide dismutase
mPTP	mitochondrial permeability transition pore
mtDNA	mitochondrial DNA
mtROS	mitochondrial ROS
ROS	reactive oxygen species
SHRSP	spontaneously hypertensive stroke-prone rats
SIRT3	sirtuin 3
SOCE	store-operated calcium entry
TCA	tricarboxylic acid
TSPO	peripheral benzodiazepine receptor
VDAC	voltage-dependent anion channel
